# Estimating survival and adoption rates of dependent juveniles

**DOI:** 10.1002/ece3.9005

**Published:** 2022-06-16

**Authors:** Phillip A. Street, Thomas V. Riecke, Perry J. Williams, Tessa L. Behnke, James S. Sedinger

**Affiliations:** ^1^ 6851 Department of Natural Resources and Environmental Science University of Nevada Reno Reno Nevada USA; ^2^ 30846 Swiss Ornithological Institute Lucerne Switzerland; ^3^ 6851 Program in Ecology, Evolution, and Conservation Biology University of Nevada Reno Reno Nevada USA

**Keywords:** adoption, brood amalgamation, hierarchical, sage‐grouse, survival

## Abstract

Population growth and fitness are typically most sensitive to adult survival in long‐lived species, but variation in recruitment often explains most of the variation in fitness, as past selection has canalized adult survival. Estimating juvenile survival until age of independence has proven challenging, because marking individuals in this age class may directly affect survival. For Greater Sage‐grouse, uniquely marking juveniles in the first days of life likely results in adverse effects to survival, detection of juveniles is not perfect, and females adopt juveniles from other parents. These challenges are encountered by researchers studying avian and mammalian species with similar life histories, yet methods do not exist that explicitly estimate all these components of the recruitment process. We propose a novel data collection method and demographic model to simultaneously estimate rates of detection, survival, and adoption of juvenile individuals. Using multiple cameras to film the beginning of juvenile activity on specific days, we obtained counts of juveniles associated with marked females. Increases of juveniles to broods provided information that enabled us to estimate rates of adoption that can be applied at the population level. Losses from broods informed apparent survival. These losses could be attributed to death, or they could be chicks that were adopted by other females. We found evidence that apparent survival of juveniles was influenced by localized weather patterns when chicks were young. Similarly, we found that young chicks were more susceptible to the adverse effect of attending females being flushed by an observer. Both of these patterns diminished quickly as chicks aged. We provide the first‐ever estimates of interval‐specific adoption rates. Our results suggest that researchers should be cautious when designing studies to estimate juvenile survival. More importantly, they provide insight into adoption, a behavior that has been known to exist for decades.

## Graphical abstract

We propose a novel data collection and an analytical method for simultaneously estimating detection, rates of adoption, and survival of juvenile Greater Sage‐grouse. We found daily weather and juvenile age to be the most influential covariates on juvenile survival. We estimated the first‐ever daily rates of adoption for this species.

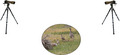

## 1 INTRODUCTION

A common goal of basic and applied research is to identify vital rates important to fitness and the persistence of populations. Understanding how a species' life history has evolved can assist investigators when predicting how populations will respond to changing environments (Cooch et al., 2001; Doherty et al., 2004; Rotella et al., 2012). In a given species, some life‐history traits evolve to be robust to environmental variation (Boyce et al., 2006; Pfister, 1998), whereas other traits evolve to be plastic in the face of environmental variation (Koons et al., 2009). Through natural selection, the combination of these traits results in phenotypes that maximize fitness, even though they may not enhance population persistence (Stearns, 1989). When selective pressure changes, the plasticity of these vital rates determines whether the population will persist.

For long‐lived species with slow generation times, variation in adult survival is generally minimal compared with the variation in reproduction (Rotella et al., 2012). If finite population growth rate for phenotypes is a reasonable surrogate for fitness, population matrix models allow researchers to explore hypotheses about selective pressure on life‐history strategies (Caswell, 2006). Doherty et al. (2004) demonstrate that these models do not always support general hypotheses based on life‐history theory. If erroneous estimates of vital rates are used, or if important vital rates are not considered, then these types of studies are inadequate to predict how populations will respond to environmental variation.

Estimation of survival from birth to parental independence and ultimately recruitment into the breeding population has been a focus of population biologist for decades (Mayfield, 1961; Williams et al., 2020). Methods to estimate survival in this age class often rely on individually marked juveniles (Gregg & Crawford, 2009). For many species, however, marking young individuals is not feasible because marks can negatively impact survival (Hastings et al., 2009), or it is difficult to apply marks that allow for growth. An alternative to marking individual juveniles is to count the offspring dependent on uniquely identifiable parents to estimate survival (Flint et al., 1995; Lukacs et al., 2004; Williams et al., 2020). The analytical methods available to estimate survival from these types of data either assume that offspring can be observed perfectly (Flint et al., 1995; Manly & Schmutz, 2001) or that the number of offspring do not increase after the initial count (Lukacs et al., 2004; Williams et al., 2020).

In some species, if the cost of parental care per sibling is low, alloparental care may arise if it is beneficial to the survival of offspring of the adopting parent (Eadie et al., 1988; MacLeod & Lukas, 2014). Riedman (1982) documented alloparental care and adoption of young in 120 mammalian species and 150 avian species. Avian species classified as *Galliformes* and *Anatidae* share an ancient ancestry in the clade *Galloanserae* (Hackett et al., 2008; Winkler et al., 2015), and many species exhibit alloparental care. Offspring from both groups are precocial, and the cost of adoption to the survival of attending parents is low compared with altricial species, although there may be costs associated with future reproductive attempts (Leach et al., 2019). Adoption has been documented in several species of *Galliformes*, including Northern Bobwhites (Faircloth et al., 2005), rock and white‐tailed ptarmigan (Wong et al., 2009), and is common in waterfowl (Beauchamp, 1997; Eadie et al., 1988; Manly & Schmutz, 2001). These adoptions violate the assumption of closure required by current analytical methods.

We use Greater Sage‐grouse (*Centrocercus urophasianus*, hereafter sage‐grouse) as an example of a novel approach for estimating apparent pre‐fledging survival and the rate of adoption into broods. Survival estimates of sage‐grouse chicks from hatching to independence are substantially less frequent than estimates of nest success (Gibson et al., 2015; Smith et al., 2019) even though pre‐fledging survival may be more variable than nest success. In years with more fall and winter precipitation, evidence suggests sage‐grouse lay larger clutches (Blomberg et al., 2014). Chick survival may also be higher in years with more cumulative winter precipitation because the herbaceous understory responds positively to more moisture, and sage‐grouse rely on grasses and forbs for energetic demands as well as for cover (Gibson et al., 2017; Wann et al., 2020). We suggest that, as a result of the relative lack of attention to this vital rate, the role of pre‐fledging survival in population dynamics and individual fitness has been under‐appreciated in sage‐grouse (Dahlgren et al., 2016; Taylor et al., 2012) and other species with precocial young (Acevedo et al., 2020; Cooch et al., 2001).

Estimation of juvenile survival has been hampered by three main constraints (1) chicks are too small to uniquely mark without affecting survival (Davis et al., 2016; Gregg & Crawford, 2009); (2) chicks cannot be detected with a probability of one (Gibson et al., 2016; Riley & Conway, 2020; Riley et al., 2021); (3) brood amalgamation makes it impossible to follow unmarked or marked chicks through space and time, even when the identity of the attending parent is known (Dahlgren et al., 2010). Marking chicks requires capturing them and often, performing surgery to implant or attach the transmitter with sutures, resulting in uncertainty about whether death in the first days of life was due to the handling process or environmental factors (Burkepile et al., 2002; Dahlgren et al., 2010; Davis et al., 2016; Gregg & Crawford, 2009). An additional challenge is that an observer has to be within a few hundred meters of a brood to detect a signal from micro‐transmitters attached to chicks, and as a result, many chick's fates are unknown. Chicks with unknown fates could have either lost their transmitter, died, or been adopted by another female. Dahlgren et al. (2010) attempted to follow marked chicks every 1–2 days. Despite this sampling effort, roughly 18% of the chicks had unknown fates. Nevertheless, the authors observed gains of unmarked chicks in nearly half of all focal broods, and 21% of marked chicks were adopted by another female. One alternative method including plasticine‐filled bands requires recapture of marked individuals and reduces the survival of juveniles (Amundson & Arnold, 2010), while passive integrated transponders (PIT tags) require being within about a meter of an antenna (Nicolaus et al., 2008), which is impractical for free‐ranging broods.

**FIGURE 1:**
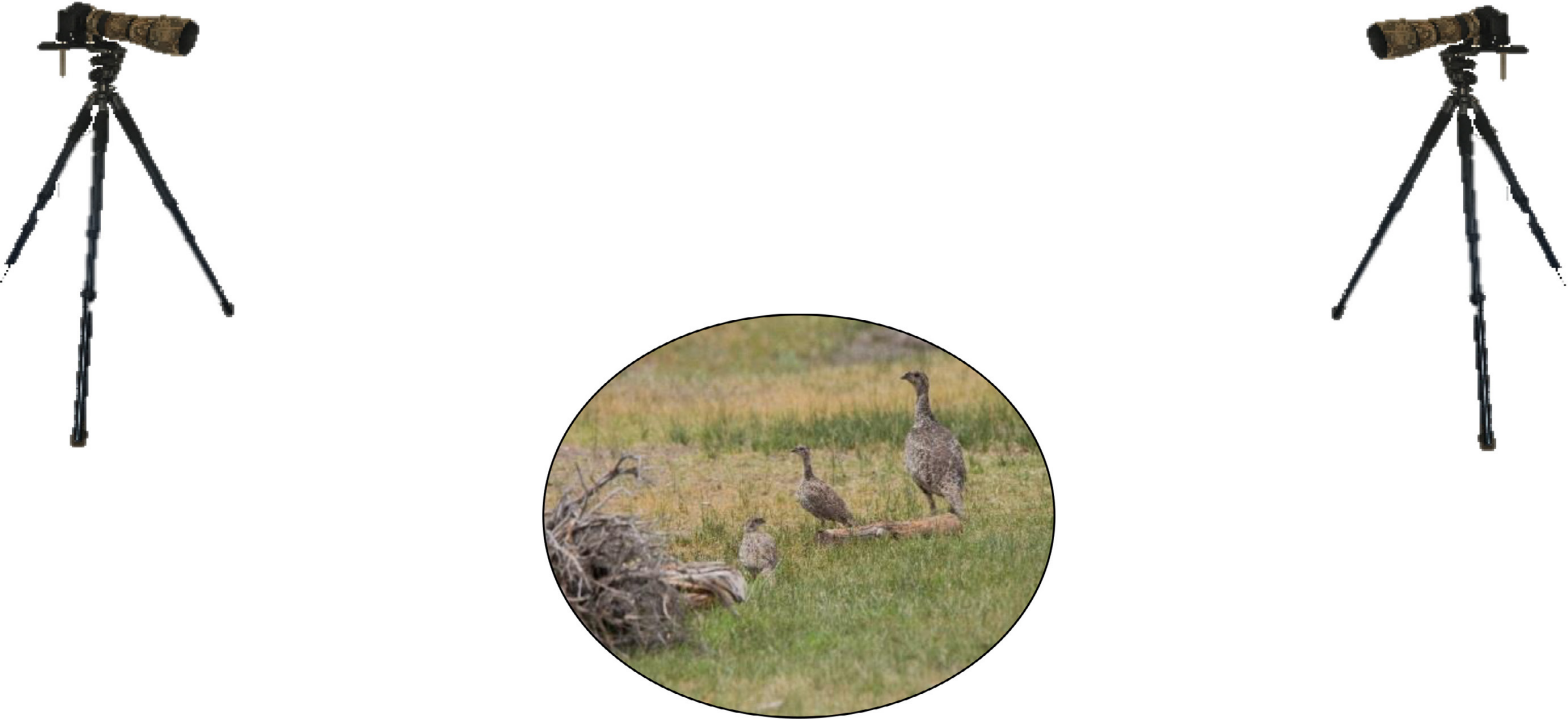
Example of camera setup to obtain counts of chicks. It is important to place cameras so that video is recorded from different angles to obtain a different field of view when conducting the counts. For example videos view https://doi.org/10.5061/dryad.0zpc8670w. The videos in the links have been shortened for smaller file size and ease of viewing. The cameras started recording before sunrise to ensure that the female was brooding her clutch. These two videos were chosen as an example of how a juvenile could have been missed using a video count. They are recordings of the same female and brood simultaneously, as described in the methods. Female 107 is an example of a female experiencing unfavorable weather conditions with newly hatched chicks. This video starts just after sunrise, as snow is beginning to fall. This first part of the video is a time‐lapse over ∼4 h as snow falls on a brooding hen. During this time period, the juveniles would be foraging if weather conditions were favorable. One juvenile comes out for a short period of time, but returns underneath the female. The video is played in real time once the female stops brooding

We developed a generalizable Bayesian hierarchical model designed to estimate juvenile survival and rates of adoption when detection is imperfect. In addition, we developed a novel data collection technique that used multiple observers equipped with remote video cameras (Figure 1) to improve the observation process. We collected data that allowed for the estimation of detection, apparent survival, and adoption while minimizing disturbance to the brood, based on a count‐based metapopulation model (Dail & Madsen, 2011). Like many precocial species, behavior of the attending sage‐grouse female changes when the entire brood is lost (Patterson, 1952). Brooding females are less likely to flush than non‐brooding females, and often perform displays to attract predators to themselves rather than their chicks. We use this information in our model to help differentiate zero counts resulting from imperfect detection, from a true zero count. This model allows investigators to examine hypotheses about factors influencing survival, including environmental as well as individual variation. We present the first‐ever estimates of adoption controlled for detection and survival of offspring.

## 2 METHODS

### 2.1 Study area

We collected sage‐grouse reproduction data from three study areas within the Great Basin, Northern Nevada and Southern Oregon, USA, from 2013 to 2018. Hart and Sheldon are National Wildlife Refuges created with the goal to preserve wildlife for future generations. The last study area, Massacre, is managed for multiple use, including livestock and feral horse grazing. Gridded annual precipitation and mean temperature data downloaded from the Climate Engine online interface (Abatzoglou, 2013; Huntington et al., 2017) were similar for the three study areas. Mean annual precipitation from 1979 to 2019 was 332, 334, and 268 mm for Hart, Massacre, and Sheldon, respectively, while mean temperature was 7.01, 7.57, and 6.93°C. Precipitation occurred primarily during winter and spring months followed by a dry summer period.

### 2.2 Clutch size, chick survival, and adoption

We captured female sage‐grouse by spotlighting (Giesen et al., 1982) during the breeding season from mid‐March through early May. Females were also captured in the fall (August through November) to supplement spring captures. All captured sage‐grouse received a metal band with a unique identifying number and were fitted with a 22 g VHF radio‐collar. After release, we monitored females until death or collar failure. From March to June, we located females twice a week by ground telemetry to determine nesting status. We checked females on nests twice a week until the fate of the nest could be determined: hatched, depredated, or abandoned.

For each female that successfully hatched a nest resulting a brood (*i*), we counted the number of hatched eggs ($c_{i,a=1,k}$) with detached membranes to estimate how many chicks were present at hatch $N_{i,a=1,k}$ when their age (*a*) was equal to day 1. If the number of hatched eggs was not clear, then this value was considered unknown. To model these unknown values, mean brood size at hatch (*λ*) was modeled for each female, as $N_{i,a=1,k}∼\text{Poisson}\left(\lambda_{i,k}\right)$. If the number of hatched eggs could be reliably determined ($c_{i,a=1,k}$), we assumed there was no error in the count $c_{i,a=1,k}=N_{i,1,k}$. To assess weather effects, we fit a linear model as $\log\left(\lambda_{i,k}\right)=\beta_{\lambda ,0}+\beta_{\lambda ,\text{winter}}\left({\text{winter}}_{i}\right)+\varepsilon_{\lambda ,k}$, where winter was the total amount of precipitation that fell between December 1 and March 1 at each nest site and $\varepsilon_{\lambda ,k}$ was the deviation from the weather model intended to assess ecological effects associated with year and site. Each site and year (*k*) were considered to be discrete random variables with 16 different categories. Each brood was assigned to one site–year category (e.g., Hart 2015; Sheldon 2014). We modeled these as random deviations from a normal distribution ($\varepsilon_{\lambda ,k}∼\text{normal}\left(0,\sigma_{\lambda }^{2}\right)$) with a common variance $\sigma_{\lambda }^{2}$, were $\sigma_{\lambda }$ had a uniform prior distribution between 0 and 5.

We hypothesized that chick survival would be lowest within the first week. To help assess this hypothesis, we attempted to get an additional count of chicks within three days of hatch, after which, we attempted to obtain a count every week. To minimize disturbance of the brood and maximize detection of chicks, we devised a novel observation method using remotely placed video cameras (Figure 1). Observers worked in teams of two to track radio‐marked females just before sunrise to ensure that the females were brooding their chicks. Observers then attempted to get a visual location without flushing the bird. If successful, a camera was placed where we first obtained a visual of the female and recording was started. Observers then placed a second camera so that it recorded the female from a different angle. Observers left cameras in place and retreated to a location that was perceived to be no longer detectable by females. Observers monitored the female from a distance using radio telemetry and retrieved cameras after females and chicks had left the location. Counts of chicks associated with each female were obtained from videos. Observers reviewed the video the day of the count to make sure a count was captured by the video. Video was recorded at a minimum resolution of 720 p by multiple makes of cameras. We recommend a minimum of a 40× optical zoom. If a brooding female was accidentally flushed while trying to obtain a visual location, observers independently counted the number of chicks before they dispersed. We stored counts in an array $\left(c_{i,a,j,k}\right)$, where *i* was the unique female, *a* was age of the brood, *j* indexed either the camera or observer involved in the multiple count, and *k* was the site and year the female was monitored. We modeled detection probability $\left(p_{i,a}\right)$ for each observer, as $c_{i,a,j,k}∼\text{Binomial}\left(p_{i,a},N_{i,a,k}\right)$. There were two covariates that we hypothesized would influence detection, including how the count was obtained (flushed or camera) and age of the brood. However, in early exploratory models we found little support for an effect of either of these covariates on detection. As such, we chose to model detection as a constant rate.

Brood size can change from one age to the next in three ways; chicks can die, chicks can be adopted by another female, or chicks from another female can be adopted into the focal female's brood. From an observation perspective, we could only observe losses or additions in a focal brood and could not differentiate whether a chick that disappeared was adopted by another female or if the chick died. Nevertheless, we could estimate the net number of chicks that survived and remained with their original mother (were not adopted by another female) from one age to the next $\left(N_{i,a,k}^{\text{survival}}\right)$ by estimating apparent survival $\left(\phi_{i,a,k}\right)$. The number of chicks adopted into the brood $\left(N_{i,a,k}^{\text{adopt}}\right)$ could be informed by observed gains in the brood. If a female lost all of her brood, we assumed she would not adopt chicks from another brood. Thus, the model statement for these parameters was as follows:

(1) $$\begin{array}{c} N_{i,a,k}=N_{i,a‐1,k}^{\text{survival}}+N_{i,a‐1,k}^{\text{adopt}},\;a=2,\ldots ,42\\ N_{i,a‐1,k}^{\text{survival}}∼\text{Binomial}\left(\phi_{i,a‐1,k},N_{i,a‐1,k}\right),\\ N_{i,a‐1,k}^{\text{adopt}}∼\left\{\begin{array}{cc} \text{Negative}\;\text{Binomial}(r,h_{i,a‐1}), & N_{i,a‐1}>0\\ 0, & N_{i,a‐1}=0, \end{array}\right.\\ h_{i,a}=\frac{r}{r+\mu_{i,a}} \end{array}$$

where r was the scale parameter of the Negative Binomial and $\mu_{i,a,k}$ was the mean number of chicks adopted in the interval. Female behavior was an additional piece of information that we used to estimate $\left(\phi_{i,a,k}\right)$. All counts began while the female should have been brooding her chicks (i.e., before sunrise). At this time of day, when a female had a brood (at least one chick), she was much less likely to flush when an observer was present. If a female with a brood did flush, she usually remained close to the brood, often intentionally making herself visible, and sometimes performed a broken wing display (Patterson, 1952). When a female did not have a brood, she typically flushed in the presence of an observer, often flying long distances, and never made herself visible after flushing. Observers would rush to the spot where the female should have been brooding her chicks and searched for any chicks that could have retreated into the vegetation for cover. If the observer found no chicks, we used this zero count in combination with the female's behavior to determine whether the brood had been lost entirely. Early within the first year of the study, we relocated each female the following morning to verify that the brood had been lost. We found that we were able to determine whether an entire brood had been lost with 100% certainty and eliminated the protocol for a follow‐up check. If for some reason the observer was uncertain about the fate of the brood, a recount was attempted the following morning. The most common reason for this uncertainty was the observers arriving after sunrise or at a time when the female was not brooding. We used these diagnostic, consistent behaviors, in combination with zero counts to discern whether females had a brood. We took advantage of this partially observable latent state $\left(z_{i,a,k}\right)$ to model the probability that at least one chick survived and remained with the original female $s_{i,a,k}^{\text{brood}}$, known in the literature as brood survival (Dzus & Clark, 1998; Fields et al., 2006). $z_{i,a,k}$ was 1 if the female was known to have a brood, and 0 if we determined the female had lost her brood based on behavior. We considered $z_{i,a,k}$ to be unknown between the last time the female was located with a brood and when she was located without a brood or if brooding status could not be determined. Brood survival was linked to apparent chick survival as:

(2) $$\begin{array}{c} z_{i,a,k}∼\left\{\begin{array}{cc} \text{Bernoulli}\left(s_{i,a,k}^{\text{brood}}\right), & z_{i,a‐1,k}=1\\ 0, & z_{i,a‐1,k}=0, \end{array}\right.\\ s_{i,a,k}^{\text{brood}}=1‐\left(1‐\phi_{i,a,k}^{N_{i,a‐1,k}}A_{i,a,k}\right) \end{array}$$

where $A_{i,a,k}$ is the probability of adopting at least one chick derived from the Negative Binomial distribution as ${\frac{r}{r+\mu_{i,a}}}^{r}$ with the parameters *r* and *μ*.

We explored hypotheses about apparent chick survival related to weather and study areas as,

$$\begin{array}{c} \begin{array}{cc} \mathrm{logit}(\phi_{i,a,k}) & \;=\beta_{\phi ,0}+\beta_{\phi ,\mathrm{age}}\left({\mathrm{age}}_{i,a}\right)+\beta_{\phi ,\mathrm{winter}}\left({\mathrm{winter}}_{i}\right)\\ & \;+\beta_{\phi ,\mathrm{flush}}\left({\mathrm{flush}}_{i,a}\right)+\beta_{\phi ,\mathrm{tmin}}\left({\mathrm{tmin}}_{i,a}\right)+\beta_{\phi ,\mathrm{ppt}}\left({\mathrm{ppt}}_{i,a}\right)\\ & \;+\beta_{\phi ,\mathrm{tmin},\mathrm{age}}\left({\mathrm{tmin}}_{i,a}\right)\left({\mathrm{age}}_{i,a}\right)+\beta_{\phi ,\mathrm{ppt},\mathrm{age}}\left({\mathrm{ppt}}_{i,a}\right)\left({\mathrm{age}}_{i,a}\right)\\ & \;+\beta_{\phi ,\mathrm{flush},\mathrm{age}}\left({\mathrm{flush}}_{i,a}\right)\left({\mathrm{age}}_{i,a}\right)+\varepsilon_{\phi ,k} \end{array}, \end{array}$$

where ${\mathrm{tmin}}_{i,a}$ was a daily minimum temperature, ${\mathrm{ppt}}_{i,a}$ was a measure of daily precipitation, ${\mathrm{winter}}_{i}$ was the total amount of precipitation that fell between December 1 and March 1 at each nest site, and ${\mathrm{flush}}_{i,a}$was a matrix with a 1 if the count was obtained using a video camera, and 0 if it was by an observer. All the weather covariates were downloaded from PRISM at a 4 km resolution (Daly et al., 1994). We modeled additional variation associated with site and year as a random deviation from a normal distribution ($\varepsilon_{\phi ,k}∼\text{normal}\left(0,\sigma_{\phi }^{2}\right)$) with a common variance $\sigma_{\phi }^{2}$, where $\sigma_{\phi }$ had a uniform prior distribution between 0 and 5. Because females with broods were not stationary on the landscape, the covariates became location‐specific indexed by age. Because females were not located every day, we assumed that females were stationary on the landscape until they were located again.

Because adoption occurred infrequently, we were limited by the data in the number of hypotheses we could test related to adoptions. We did model site and year variation in adoption as $\log\left(\mu_{i,a,k}\right)=\beta_{\mu ,0}+\varepsilon_{\mu ,i}$, where $\varepsilon_{\mu ,i}$ were random deviations from a normal distribution ($\varepsilon_{\mu ,i}∼\text{normal}\left(0,\sigma_{\mu }^{2}\right)$) with a common variance $\sigma_{\mu }^{2}$, where $\sigma_{\mu }$ had a uniform prior distribution between 0 and 5. $N_{i,42,k}$ was the number of chicks born to each female that survived to 42 days and were not adopted out of the brood as well as the total number of chicks that each female adopted. If we assume that our marked hens are representative of the entire population, then an intuitive measure of true survival from hatch to fledging is $S^{42,k}=N_{i,42,k}/N_{i,1,k}$.

The estimated parameters requiring prior distributions included $\beta_{p},\;\beta_{\lambda },\;\beta_{\phi },\;\beta_{\mu }$, and $\beta_{r}$, where β were the coefficients of a linear model associated with each parameter: p,λ,ϕ,μ,r. We used a logit link to constrain the linear model for *p* and ϕ to be between 0 and 1. For the prior distributions on $\beta_{p},\;\beta_{\phi }$ we used a normal distribution with a mean of 0 and a variance equal to $1.5^{2}$(Northrup & Gerber, 2018). We used a log link to constrain the linear model for λ, μ, and *r* to be greater than 0. For the prior distributions on $\beta_{\lambda ,0}$ and $\beta_{\lambda ,\text{winter}}$, we used a normal distribution with a mean of 0 and a variance equal to 1.5^2^. Because adoption of sage‐grouse occurred infrequently when measured at a daily interval, we constrained the prior distribution of $\beta_{\mu }\;$and $\beta_{0,r}$ to a normal distribution with a mean of −1 and a variance of 1. Together, these priors resulted in a distribution of the number chicks being adopted into a brood per day with a mean of 0.6305, and a 97.5% quantile of 4. The joint Bayesian posterior distribution for our hierarchical model was

(3) $$\begin{array}{l} {}[N_{1},\lambda ,\beta_{\lambda },\sigma_{\lambda }^{2},N_{a},N_{1,\ldots ,41}^{\text{survival}},\phi_{a},\beta_{\phi },\sigma_{\phi }^{2},N_{1,\ldots ,41}^{\text{adopt}},\mu_{a},\beta_{\mu },\sigma_{\mu }^{2},r,Z_{2,\ldots ,42},p_{a},\beta_{p}|C_{1},C_{2,\ldots ,42}]\propto \\ \prod_{i=1}^{n}\prod_{a=r_{i}}^{A}[C_{2,\ldots ,a}|N_{1,\ldots ,41}^{\text{survival}},N_{1,\ldots ,41}^{\text{adopt}},p_{i,a}]\times \\ \;[N_{i,1,\ldots ,a‐1}^{\text{survival}}|\phi_{i,a‐1},N_{i,a‐1}]\times \\ \;{\left[N_{i,1,\ldots ,a‐1}^{\text{adopt}}|r,\mu_{i,a‐1},N_{i,a‐1}\right]}^{I_{\left\{N_{i,a‐1}>0\right\}}}I_{\left\{N_{i,a‐1}>0\right\}}^{\left(1‐I_{\left\{N_{i,a‐1}^{\text{adopt}}=0\right\}}\right)}\times \\ \;[z_{i,a}|z_{i,a‐1},\phi_{i,a‐1},N_{i,1,\ldots ,a‐1}^{\text{survival}},N_{i,1,\ldots ,a‐1}^{\text{adopt}},r,\mu_{i,a}]\times \\ \;\left[\lambda |\beta_{\lambda },\sigma_{\lambda }^{2}\right]\left[\phi |\beta_{\phi },\sigma_{\phi }^{2}\right]\left[\mu_{i,a}|\beta_{\mu },\sigma_{\mu }^{2}\right]\times \\ \;\left[N_{1}\right]\left[r\right]\left[\beta_{\lambda }\right]\left[\sigma_{\lambda }^{2}\right]\left[\beta_{\phi }\right]\left[\sigma_{\phi }^{2}\right]\left[\beta_{\mu }\right]\left[\sigma_{\mu }^{2}\right]\left[\beta_{p}\right] \end{array}$$

Models were run with 3 chains, with a total of 60,000 iterations each with a burn‐in period of 40,000 saving every other iteration using JAGS (Plummer et al., 2003) and the JAGsUI package (Kellner et al., 2019). Convergence of the parameters was assessed using the $\hat{R}$ statistic, as well as visual inspection of the chains (Gelman et al., 2013). For all estimated and derived parameters, we report the mean and the posterior standard deviation. For all β values of effect size, we report the proportion of the posterior greater than or less than 0 (*F*) and consider a value greater than 0.95 to be a meaningful effect.

### 2.3 Data simulation

To assess model performance, we simulated data using Equation (3), with known parameter values representative of many wildlife applications and fit our model of the number offspring born, offspring survival, adoption, and detection. The estimated posterior distributions were evaluated to determine the ability our model to recover the generated parameter values as well as the presence of any bias. We simulated 96 datasets, each containing 100 females with offspring. We chose to use parallel processing to conduct our simulations. We decided to simulate 96 datasets because we had 8 computer cores available to conduct the simulation. Thus, each core simulated and analyzed 12 datasets. We used predetermined distributions to obtain different parameter values to generate each dataset (Table 1). We built four constraints into the simulations. First, we forced daily offspring survival to be high and to increase with age, thus $\text{logit}\left(\phi_{i,t}\right)=\beta_{\phi ,0}+\beta_{\phi ,\text{age}}$ where $\beta_{\phi ,0}$ was randomly drawn from a normal distribution with a mean of 1.6 and a variance of $0.2^{2}$ and $\beta_{\phi ,\text{age}}$ was drawn from a normal distribution with a mean of 0.05 with a variance of 0.02. Second, we forced adoptions to occur infrequently and range from 1 to 5; hence, we choose to simulate the adoption process using a negative binomial distribution with a small mean $\left(\log\;\text{normal}\left(‐1.4,0.4\right)\right)$ and a small rate $\left(\log\;\text{normal}\left(‐1,0.4\right)\right)$. Third, given the complexity of this model, we limited our simulation to have high detection probabilities randomly drawn between 0.8 and 1. Lastly, the number of chicks in the total simulated population at 42 days of age could not exceed the number of chicks born. If the simulated dataset violated this constraint, it was discarded and a new dataset was simulated and analyzed. Patterns in the simulated data were consistent with our own experience and that of others (Gibson et al., 2017). For each dataset generated, we fit our model and evaluated the proportion of times the 95% credible intervals overlapped the true parameter values (Little, 2006; Williams & Hooten, 2016) and calculated mean bias (true parameter—mean of the posterior distribution) for each parameter.

**TABLE 1:** Parameters and the distributions used to generate known values to simulate 96 different datasets

Parameter	Simulation	Prior	Coverage (%)	Mean bias
$s^{42}$	$\frac{N_{i,42}}{N_{i,1}}$	$\frac{N_{i,42}}{N_{i,1}}$	94	0.003
$N_{i,1}$	Poisson(6)	lognormal(0,1.5)	97	0
$\beta_{\phi ,0}$	$\text{normal}\left(1.6,0.2\right)$	$\text{normal}\left(0,1.5\right)$	96	0.007
$\beta_{\phi ,\text{age}}$	$\text{normal}\left(0.05,0.02\right)$	$\text{normal}\left(0,1.5\right)$	84	0.004
μ	$\log\;\text{normal}\left(‐1.4,0.4\right)$	$\log\;\text{normal}\left(0,1.5\right)$	89	−0.016
*r*	$\log\;\text{normal}\left(‐1,0.2\right)$	$\log\;\text{normal}\left(0,1.5\right)$	99	0.231
*p*	$\text{uniform}\left(0.8,1\right)$	$\text{uniform}\left(0,1\right)$	93	−0.006

Note

Coverage is the percentage of times that the known value is within the 95% credible intervals of the posterior distribution. For the normal and log normal distributions, the first number is the mean, and the second number is the standard deviation.

## 3 RESULTS

### 3.1 Simulation results

**FIGURE 2:**
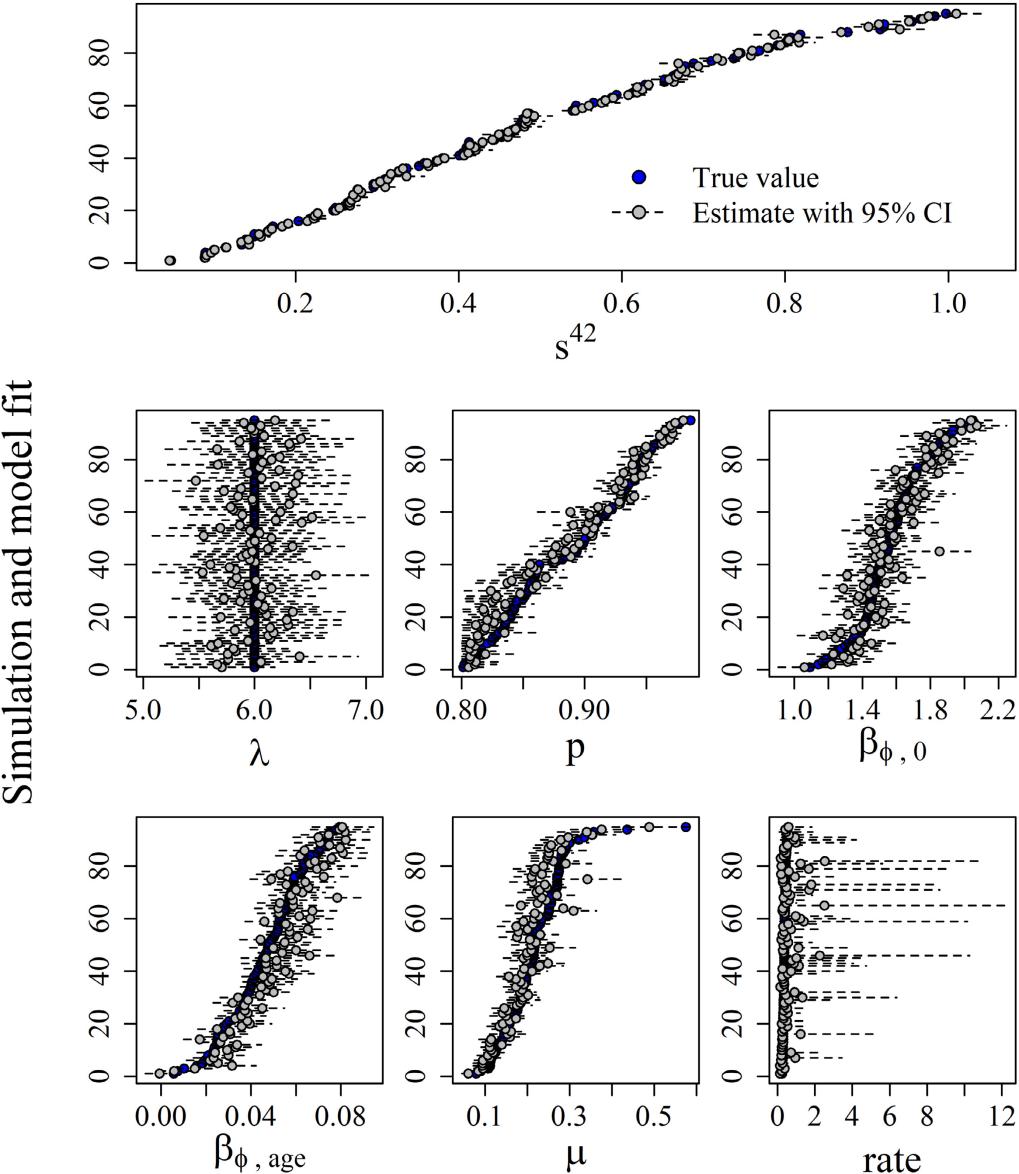
Results from 96 data simulations with the juvenile survival model fit to 6 parameters. λ is the mean of a Poisson distribution that defines clutch size at hatch. *p* is a constant detection probability. $\beta_{0}$ and $\beta_{\text{age}}$ are used to define an age specific apparent chick survival rate. μ and rate define the parameters of a negative binomial distribution associated with daily adoptions into a brood. For each graph, the simulations were sorted from smallest to largest based on the true parameter estimates to make visualization easier

Given a sample size of 100 parental adults and a weekly sampling interval, we demonstrate that all parameters were identifiable with reasonable precision (Figure 2, Table 1). The simulated parameter values were recovered by the estimated posterior distributions for λ, $\beta_{\phi ,0}$, $\beta_{\phi ,\text{age}}$, μ, *r*, and *p* a high proportion of the time, resulting in an unbiased estimate of the derived parameter of true survival with high precision (Figure 2). This parameter ranged from 0 to 1, and these results suggest that our model will work well for a variety of applications.

### 3.2 Greater sage‐grouse example

**FIGURE 3:**
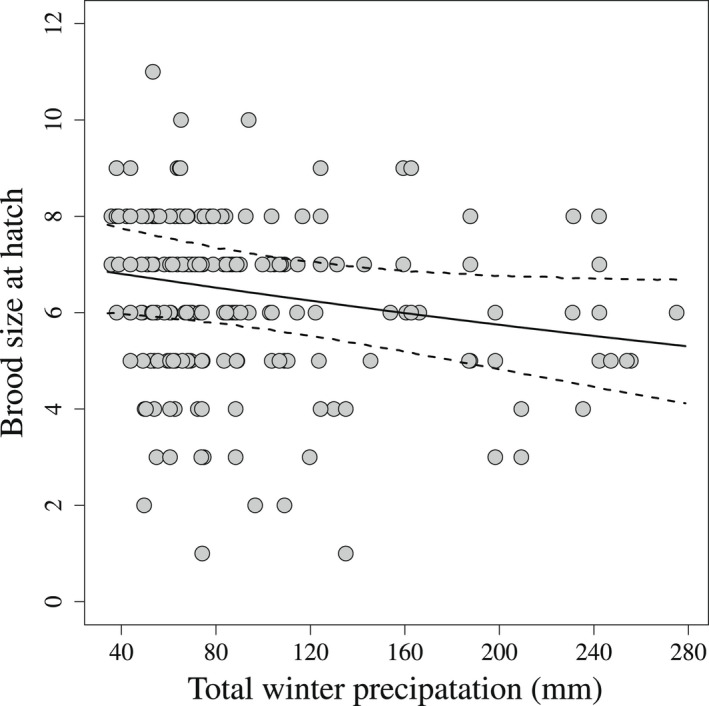
Brood size at hatch as a function of cumulative precipitation from December 1 to March 1 at the nest site. Points are the observed brood sizes for 279 nests

**FIGURE 4:**
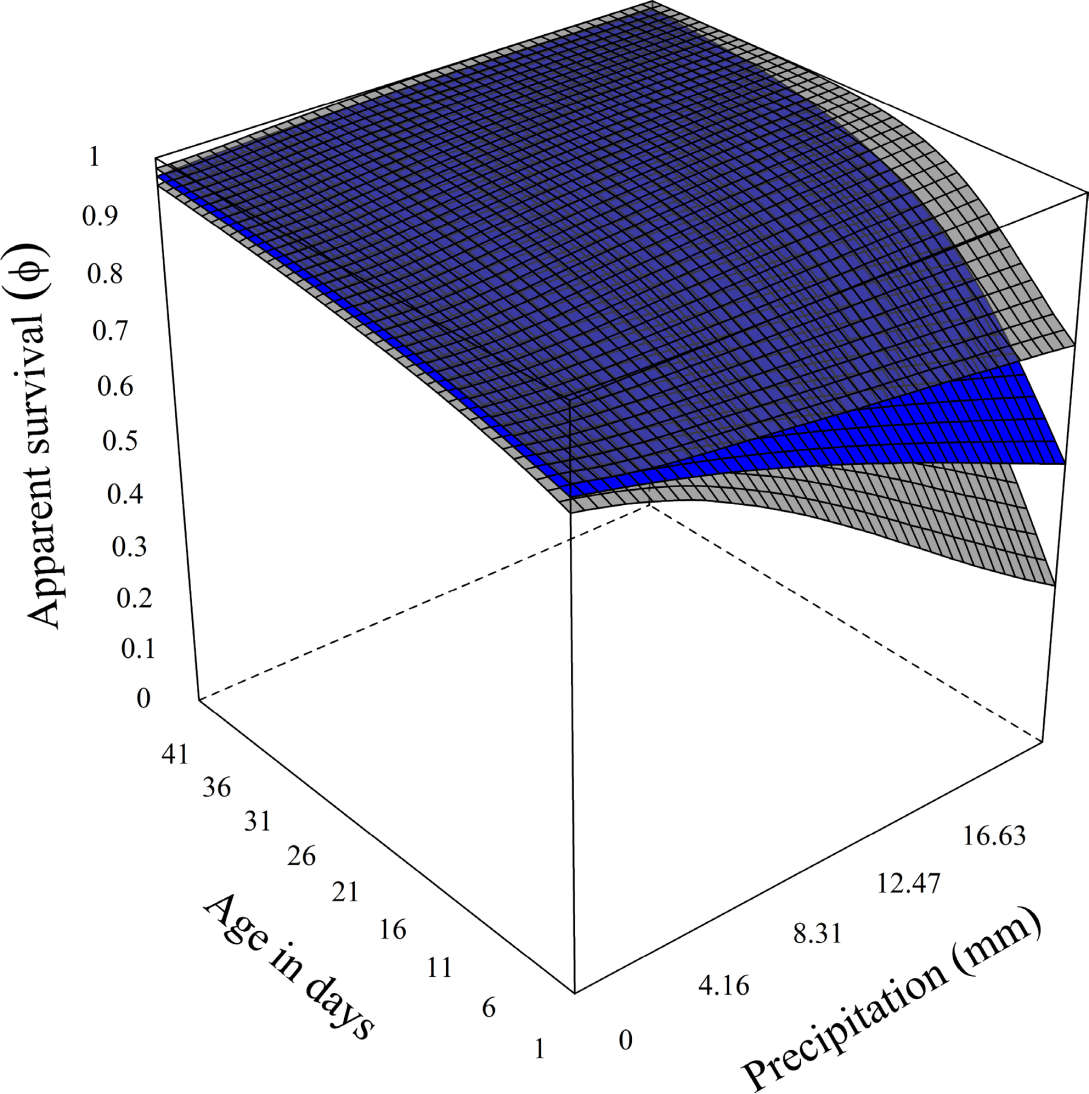
Daily probability of a chick surviving and remaining with the same female (ϕ), based on the weather they experienced and their age in days. Precipitation was the total amount for each day. The blue surface is the mean estimate, while the transparent gray surfaces represent the 95% credible intervals

From 2013 to 2018, we monitored 279 sage‐grouse broods, hatched by 240 unique females across 3 study sites. Mean brood size (λ) of Greater Sage‐grouse at hatch for nests experiencing average winter precipitation was 6.448 (SD = 0.028); however, we found evidence that females that chose nest sites with higher cumulative winter precipitation hatched smaller broods (Figure 3, $\beta_{\lambda ,\text{winter}}$ = −0.051, SD = 0.027, *F* = 0.969). After hatch, an individual chick had a mean probability of surviving day one and remaining with its original mother ($\phi_{a=1}$) of 0.854 (SD = 0.011), if there was no precipitation. If it rained or snowed, the probability of a chick surviving decreased (Figure 4, $\beta_{\text{prcp}}=‐0.133$, SD = 0.048, *F* = 0.996). As chicks aged, they survived at higher rates ($\beta_{\text{age}}$ = 0.052, SD = 0.004, *F* = 1) and the effect of precipitation diminished ($\beta_{\text{prcp,age}}$ = 0.006, SD = 0.005, *F* = 0.865). We found little evidence that temperature affected apparent survival ($\beta_{\text{temp}}$ = 0.075, SD = 0.051, *F* = 0.928) or interacted with age ($\beta_{\text{temp,age}}$ = −0.012, SD = 0.005, *F* = 0.991).

**FIGURE 5:**
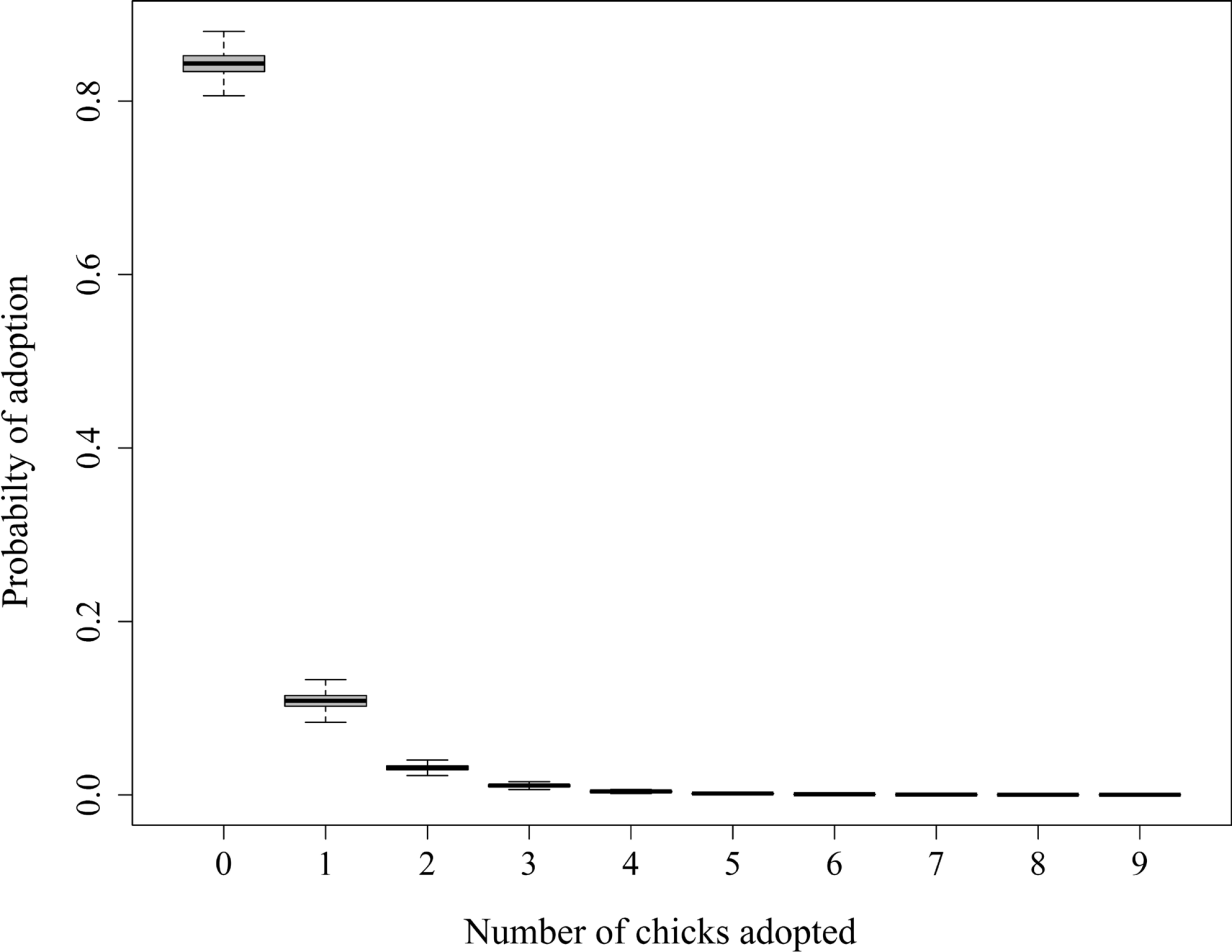
Daily probabilities of adopting the number of chicks displayed on the *x*‐axis. These estimates were informed by observed gains to a brood and modeled with a negative binomial distribution

If females had a brood, the daily probability that she would adopt at least 1 chick was (0.155, SD = 0.015). When females did adopt chicks, it was most likely a small number; 99.98% of adoption events were 3 chicks or less (Figure 5). Adoptions were modeled with a negative binomial distribution and a constant rate estimated to be 0.306 (SD = 0.064), resulting in the mean number of chicks being adopted per day of 0.226 (SD = 0.024).

**FIGURE 6:**
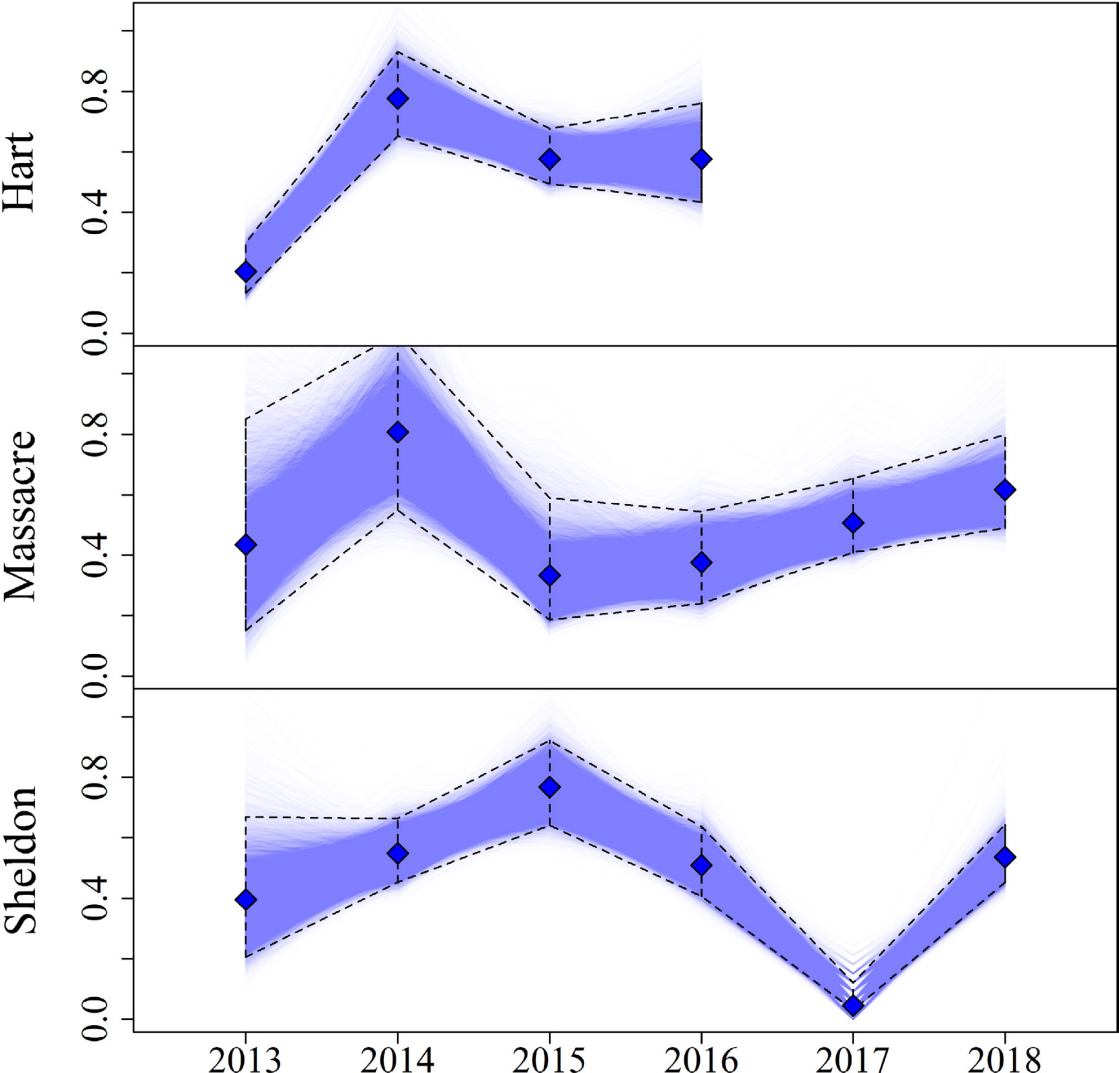
Average proportion of chicks fledged from the chicks hatched for females at each site and year combination. These estimates are for females we had marked given the weather they experienced during that year

We observed additional variation not explained by covariates among study areas and years in both apparent survival ($\sigma_{\phi }$ = 0.228) and the probability of adoption ($\sigma_{r}$ = 0.31). When combined, this random variation and the variation due to weather resulted in substantial variation in the ratio of chicks fledged to the number hatched, that is, true survival accounting for adoption (Figure 6). Probability of a chick surviving from hatch to fledging ranged from 0.047 on Sheldon in 2017 to 0.77 on Massacre in 2014 (Table 2).

**TABLE 2:** Number of broods (*n*) marked at each site for each year

Site and year	*n*	$\varepsilon_{\phi }$	SD	$\varepsilon_{\mu }$	SD	$S^{42}$	SD
Hart 2013	15	−0.261	0.139	−0.206	0.226	0.192	0.041
Hart 2014	34	0.272	0.140	0.021	0.158	0.818	0.077
Hart 2015	27	0.079	0.127	0.023	0.161	0.581	0.052
Hart 2016	11	0.163	0.149	−0.288	0.221	0.57	0.082
Massacre 2013	4	0.071	0.171	−0.179	0.307	0.452	0.174
Massacre 2014	21	−0.156	0.142	0.409	0.215	0.77	0.134
Massacre 2015	16	0.005	0.134	−0.201	0.225	0.291	0.07
Massacre 2016	15	−0.207	0.150	0.214	0.203	0.384	0.076
Massacre 2017	12	0.024	0.178	0.049	0.202	0.528	0.066
Massacre 2018	16	0.010	0.137	0.199	0.197	0.629	0.090
Sheldon 2013	8	−0.022	0.152	−0.212	0.267	0.466	0.151
Sheldon 2014	26	0.071	0.124	−0.134	0.166	0.546	0.052
Sheldon 2015	22	0.126	0.129	0.163	0.166	0.756	0.070
Sheldon 2016	13	0.199	0.146	−0.046	0.202	0.513	0.050
Sheldon 2017	11	−0.306	0.195	−0.228	0.246	0.047	0.030
Sheldon 2018	28	−0.040	0.130	0.323	0.183	0.525	0.049
	*N* = 279	$\beta_{0}^{\phi }$ = 1.65	$\sigma_{\phi }$ = 0.228	$\beta_{0}^{\mu }$ = −1.491	$\sigma_{\mu }$ = 0.310	$\mu^{S^{42}}$ = 0.504	$\sigma^{S^{42}}$ = 0.218

Note

Apparent survival (ϕ) was modeled as a linear model with a logit link and an intercept of $\beta_{0}^{\phi }$. This intercept was representative of a mean apparent survival rate under mean weather conditions over the course of our study. $\varepsilon_{\phi }$ was the random deviation from the fixed effects model of weather and juvenile age on apparent survival (ϕ). The number of chicks adopted over each interval was modeled with a Negative Binomial distribution with the mean modeled on a log link with an intercept of $\beta_{0}^{\mu }$. $\varepsilon_{\mu }$ was the random deviation from the mean adoption size for each site and year combination. $\sigma_{\phi }$ and ${\text{sigma}}_{\mu }$ were the random error modeled for apparent survival and mean adoptions rates. $S^{42}$ was mean true survival modeled for each site and year based on weather experienced by the marked females in the sample. $\mu^{s^{42}}$ and $\sigma^{S^{42}}$ was the mean true survival and the standard deviation of all females across all years based on the weather they experienced.

Detection was estimated to be high with good precision 0.945 (SD = 0.004), most likely due to the fact that counts were conducted early in the morning to ensure that females were brooding chicks. When we assessed whether there was a difference in apparent survival due to method, we found evidence that flushing the female resulted in a reduction in survival of 0.165 (SD = 0.033) when the brood was 1 day of age, but this effect disappeared by the time chicks reached 3 days of age.

## 4 DISCUSSION

We present a novel approach to data collection, and modeling demographic rates, which provides estimates of survival as well as the adoption rate of unmarked juveniles. When adoption occurs and is not accounted for when modeling survival and detection, survival estimates would likely be overestimated (Lukacs et al., 2004; Williams et al., 2020), and may result in overly optimistic population trajectories.

We present daily rates of adoption into sage‐grouse broods that suggest that the average female who successfully reared a brood adopted at least one juvenile born to a different female before fledging her brood. Females displayed risky behavior to attract predators to themselves rather than their chicks. Kin selection has been proposed as a hypothesis to explain this seemingly altruistic behavior in other species (Eadie et al., 1988; Wong et al., 2009). Andersson (2018) suggested that high rates of female philopatry could increase relatedness of sage‐grouse in an area sufficiently that kin selection might explain the adoption of apparently unrelated offspring. Sage‐grouse show high lek, nest, and brood site fidelity (Fischer et al., 1993; Gerber et al., 2019; Gibson et al., 1991), and Jahner et al. (2016) found a high degree of relatedness among individuals with similar space use, suggesting the feasibility of Andersson (2018)'s hypothesis. Alternatively, adoptions have been explained as a mechanism to reduce predation risk for one's own young (Lengyel, 2007), although this potential benefit could be traded off against costs to future reproduction for adults rearing enlarged broods (Leach et al., 2019). Regardless of the mechanism, our observation method and demographic model are the first that we know of to incorporate adoption and survival into the same likelihood and open the door for exploration of tradeoffs between these parameters.

Guttery et al. (2013) found a negative association among winter droughts and survival of sage‐grouse juveniles during the following growing season. Similarly, Gibson et al. (2017) found that juvenile survival was negatively associated with drought, but suggested that females might be able to partially mitigate these negative effects by choosing to nest in areas that receive more precipitation or moving their broods to these areas after hatch. Conversely, we found little support for a negative effect of cumulative winter precipitation on apparent survival. We were able to quantify a negative effect of daily precipitation on apparent survival when the chicks were young, especially when it was cold. The cameras allowed us to observe the behavior of chicks and females and provide some insight into a mechanism behind the effect of weather (Figure 1, Female 107 link). On a day with no precipitation, chicks ventured out from underneath the brooding female, shortly after sunrise and immediately began foraging. In contrast, when it was either raining or snowing, the female continued to brood the chicks for hours after sunrise. If the chicks did venture out, they quickly returned to the safety of the brooding female. We suspect that chicks were not surviving because they were exposed to harsh environmental conditions and limited foraging time preventing them from meeting their energetic demands.

We also found a negative effect of flushing females on apparent survival of chicks when they were young. When a female is flushed from a brood, the brood has to regroup. We observed sage‐grouse accomplishing this by chicks calling to the female with distress calls and the female responding. If there are other females in the proximate area, they may respond to the chick's distress calls (Wallestad, 1971). Thus, by flushing females, observers may be inducing brood amalgamations that would not have occurred otherwise. Another reason flushing hens could result in lower apparent survival of juveniles is that the chicks did not regroup with the female and died of exposure. The interaction between age and flushing forced estimates of apparent survival to be similar to non‐flushed birds at 3 days of age, after which estimates of flushed birds were higher. We believe the latter is an artifact of a linear model with a logit transformation with estimates close to the boundary of 1. That is, after 3 days all daily apparent survival rates were high. We suggest that if researchers plan to flush the birds, they do not do so within 3 days of hatch. Lastly, because we found evidence for a negative effect of flushing females on apparent juvenile survival, we strongly discourage future investigators from flushing females and surgically attaching radio transmitters to juveniles within the first days of life.

We provide an example that simultaneously estimates imperfect detection, survival, and adoption using counts of juveniles in a brood. Conceptually, it is easy to conceive of multiple possibilities for how a count could change or stay the same between intervals due to these three processes. For example, if a brood were observed to contain 6 chicks at age 1 and also 6 chicks at age 7, this does not mean that survival was 100% and that no adoptions occurred; that is only one of many possible ways of observing 6 chicks during both counts. One assumption that allows these parameters to be estimated is that all of these processes are assumed to be Markovian, that is, $N_{t}$ in dependent on $N_{t‐1}$. Given that these processes are being estimated simultaneously between counts, it is important to recognize that sampling correlations exist between the estimates and their uncertainties. That is, uncertainty in the survival process also results in uncertainty in the adoption process as well as our ability to accurately count the brood. These three processes have been estimated together in previous literature. For example, in a similar model, Schmidt et al. (2015) followed radio‐marked wolves to obtain counts of unmarked pack mates to model pack dynamics. What separates our model from Schmidt et al. (2015) is the integration of the female's behavior to aid in the estimation of apparent juvenile survival, adoption, detection, and brood survival, a commonly reported parameter (Fields et al., 2006; Rotella & Ratti, 1992). We show through simulation that all of our parameters are identifiable and recoverable under a range of parameters conceivable for brood dynamics and encourage future investigators to use the provided code to do the same. We build on a growing body of literature for estimating demographic processes informed by counts of unmarked individuals.

Like Royle (2004)'s *N*‐mixture model and Dail and Madsen (2011)'s extension to allow gains to the population, our model assumes closure between replicated counts that make estimation of detection possible. Conn et al. (2018) demonstrated that N‐mixture model parameter estimates are sensitive to violations of this assumption. Our data collection method ensures that this assumption is met, because counts by either multiple observers or cameras were instantaneous and independent. Another assumption of our model is that chicks are not double counted. With cameras, we ensure that this assumption is completely met. When females are flushed, the brood often flushes immediately after and counts have to be made quickly as the birds are flying away and could result in a violation of this assumption. This problem most likely occurred when counting large broods with older chicks because they are better fliers and flush from greater distances as observers approach. We explored models with age as well as count method effects on detection and adoption and found little evidence for an effect on either parameter. Thus, we believe any bias caused by violation of this assumption to be minimal.

Barker et al. (2018) suggest collecting auxiliary data to minimize bias when estimating demographic parameters from counts of unmarked individuals. We used two pieces of auxiliary data: (1) all attending adult females were radio marked, so we could follow broods through time; (2) female behavior allowed us to determine whether the entire brood had been lost or if at least one chick was present. This parameter is often referred to as brood survival (Fields et al., 2006; Rotella & Ratti, 1992). By themselves, estimates of brood survival provide little information about how many new individuals a female fledges. We also monitored daily true survival rates as defined by Flint et al. (1995). It is important to recognize that these rates can be >1 for broods when adoption occurs, so they are not strictly probabilities. We present the product of these rates to 42 days of age as a ratio of chicks fledged to those hatched. Under the assumption that the adoption of chicks into the sample population balances adoption out of the sample, the point estimate for this ratio should be ≤1.0 when averaged across the entire sample. If investigators find this point estimate to be larger than 1, it indicates that they may have marked a biased sample of females or sample size was small.

Our simulation revealed two issues that investigators should be aware of. First, when evaluating an age effect on offspring survival ($\beta_{\phi ,\text{age}}$), we observed the lowest amount of overlap between the 95% credible intervals and true simulated parameter value with an 84% recovery rate. When we evaluated the point estimates of apparent survival (mean of the posterior) for $\beta_{\phi ,\text{age}}$, we found that they were slightly biased high (0.004). One contributing factor could be that not all offspring survive; thus, there are fewer data at the end of the time frame to inform this parameter. Additionally, if offspring do survive to an older age, then their survival probability is close to the boundary of 1 and may result in difficulty estimating this parameter using a logit transformation. Second, when estimating the number of adopted chicks with the negative binomial distribution, we recovered the mean with 89% overlap between the 95% credible intervals and true simulated parameter value, but no apparent bias when comparing the point estimate to the known value. When evaluating the rate parameter associated with the mean, we observed almost perfect overlap 99%, but when comparing the point estimate (mean of the posterior) it appeared to be biased high 0.231. This is entirely due to the fact that this parameter was always close to the boundary of 0 and has the least amount of data to inform it; thus, the resulting posterior distribution always had a long right tail. We recommend inference about the adoption process be made from the negative binomial distribution as a whole, similar to Figure 5. It is important to note that any difficulties in estimating either the daily apparent survival probabilities or the daily adoptions did not result in any apparent difficulties or biases in estimating the true overall survival. This is a parameter that has been of primary interest to investigators for decades (Flint et al., 1995; Lukacs et al., 2004), and currently no analytical method exists to provide unbiased estimates for sage‐grouse.

We believe the observation method we present has the potential to be applied to other species with dependent offspring. Regardless of observation method, our modeling framework could be applied to a variety of species. As a general approach, we suggest researchers leverage biology of the species of interest in the data collection and modeling framework. Hierarchical models provide an extremely flexible tool that can leverage multiple pieces of information such as radio telemetry, female behavior, and counts of chicks within broods that can provide novel insights into ecological processes.

## AUTHOR CONTRIBUTIONS

**Phillip** **A. Street** contributed to conceptualization (lead); data curation (lead); formal analysis (lead); funding acquisition (supporting); investigation (lead); methodology (lead); writing—original draft (lead); and writing—review and editing (equal). **Thomas V. Riecke** contributed to conceptualization (equal); methodology (equal); and writing—review and editing (supporting). **Perry J. Williams** contributed to formal analysis (supporting); methodology (supporting); writing—original draft (supporting); and writing—review and editing (supporting). **Tessa L. Behnke** contributed to conceptualization (supporting); data curation (supporting); formal analysis (supporting); investigation (supporting); methodology (supporting); writing—original draft (supporting); and writing—review and editing (supporting). **James S. Sedinger** contributed to conceptualization (supporting); data curation (supporting); formal analysis (supporting); funding acquisition (supporting); investigation (supporting); methodology (supporting); project administration (lead); writing—original draft (supporting); and writing—review and editing (supporting).

## CONFLICT OF INTEREST

The authors declare no conflicts of interest.

### OPEN RESEARCH BADGES

This article has been awarded Open Data, Open Materials Badges. All materials and data are publicly accessible via the Open Science Framework at https://doi.org/10.5061/dryad.0zpc8670w.

## Data Availability

The data that support these finding are openly available at https://doi.org/10.5061/dryad.0zpc8670w; https://doi.org/10.5061/dryad.0zpc8670w (Street et al., 2022).
